# Supplementary material for: Evaluation of endogenous miRNA reference genes across different zebrafish strains, developmental stages and kidney disease models
Source: Sci Rep. 2021 Nov 24;11:22894. doi: 10.1038/s41598-021-00075-2 (PMC8613261; doi:10.1038/s41598-021-00075-2)
Supplement: Supplementary file 1 — Supplementary Information. [file 41598_2021_75_MOESM1_ESM.pdf]

Evaluation of endogenous miRNA reference genes across different zebrafish strains, developmental stages and kidney disease models

Florian Siegerist[1*], Tim Lange[1*], Anna Iervolino[1, 2], Thor Magnus Koppe[1], Weibin Zhou[3], Giovambattista Capasso[2,4], Karlhans Endlich[1], Nicole Endlich[1]

[1]Institute of Anatomy and Cell Biology, University Medicine Greifswald, Greifswald, Germany

[2]Biogem Research Institute Gaetano Salvatore, Ariano Irpino, Italy

[3]Division of Nephrology, Department of Medicine, Icahn School of Medicine at Mount Sinai, New York City, NY, USA

[4]Department of Translational Medical Sciences, University of Campania "L. Vanvitelli", Naples, Italy.

*The authors contributed equally to this work

Supplemental Material

Address for correspondence:

Prof. Dr. rer. nat. Nicole Endlich

Friedrich-Loeffler-Str. 23c

17487 Greifswald

Germany

nicole.endlich@uni-greifswald.de

Supplemental Fig. 1

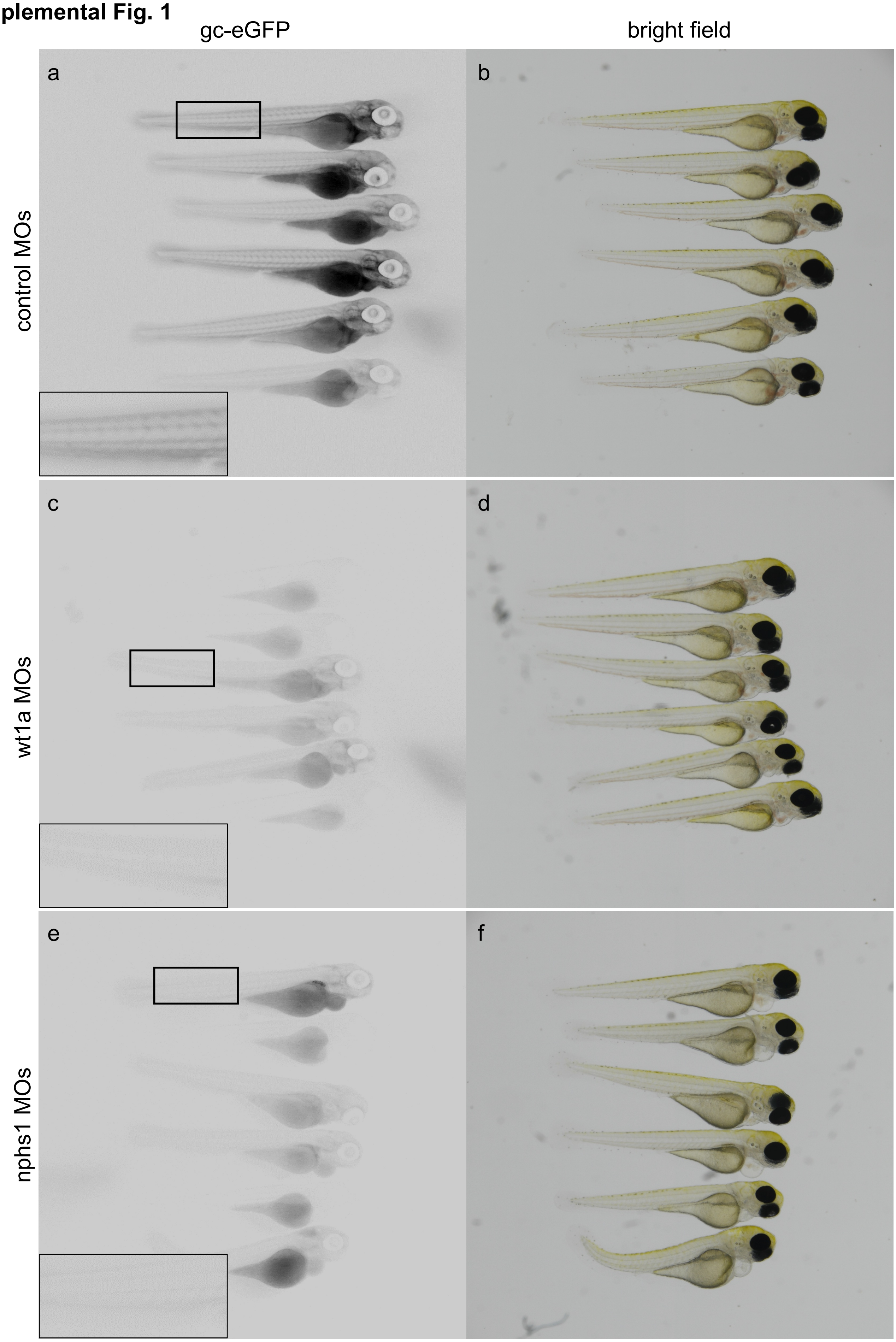

Supplementary Figure 1: Tg(fabp10a:gc-eGFP) embryos imaged at 96 hpf, show presence of the 78 kDa gc-eGFP fusion protein in the vasculature of control MO-injected larvae (a-b). In contrast to that gc-eGFP fluorescence intensity was significantly lower in *wt1a* or *nphs1* knockdown (c-f).

Supplemental Fig. 2

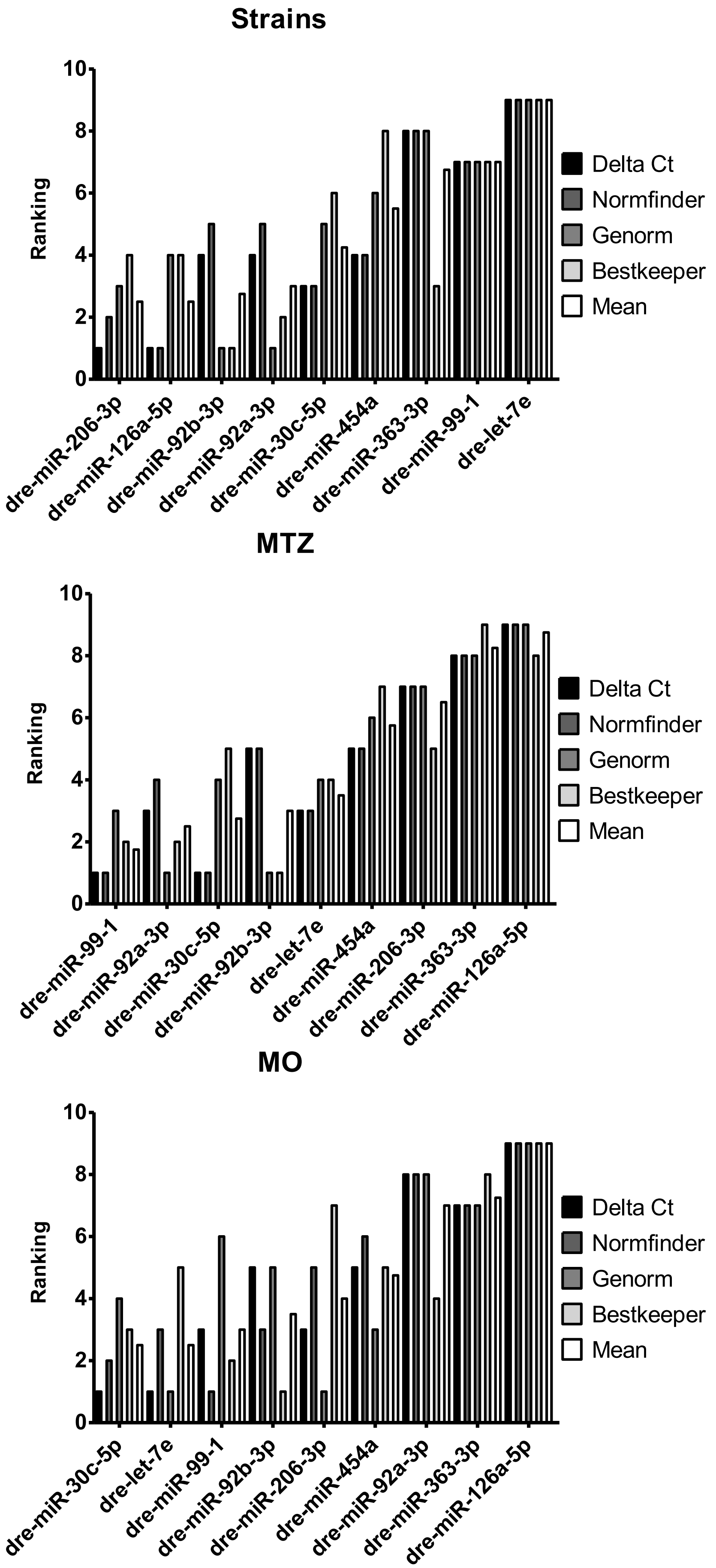

Supplementary Figure 2: Average ranking of candidate miRs in strains, MTZ and MO by 4 different normalization determination algorithms. Data was ranked by DeltaCt, Normfinder, Genorm and Bestkeeper.
